# Supplementary material for: Beyond harm’s reach? Submersion of river turtle nesting areas and implications for restoration actions after Amazon hydropower development
Source: PeerJ. 2018 Jan 8;6:e4228. doi: 10.7717/peerj.4228 (PMC5764030; doi:10.7717/peerj.4228)
Supplement: Figure S1 — Representative examples showing (A–B): potential nesting areas with suitable habitat conditions for nesting, but no nests (i.e., locations that females could use for nesting but nests were not found); (C–D): actual nesting areas (i.e., locations where females actually nested), and (E–F): unsuitable habitat areas for nesting (i.e., where substrate conditions were not appropriate for females to nest). Photo credit Fernanda Michalski. [file peerj-06-4228-s001.docx]

Figure S1: Photos characterizing *Podocnemis unifilis* nesting areas. Representative examples showing (A-B): potential nesting areas with suitable habitat conditions for nesting, but no nests (i.e., locations that females could use for nesting but nests were not found); (C-D): actual nesting areas (i.e., locations where females actually nested), and (E-F): unsuitable habitat areas for nesting (i.e., where substrate conditions were not appropriate for females to nest). Photo credit Fernanda Michalski.

| A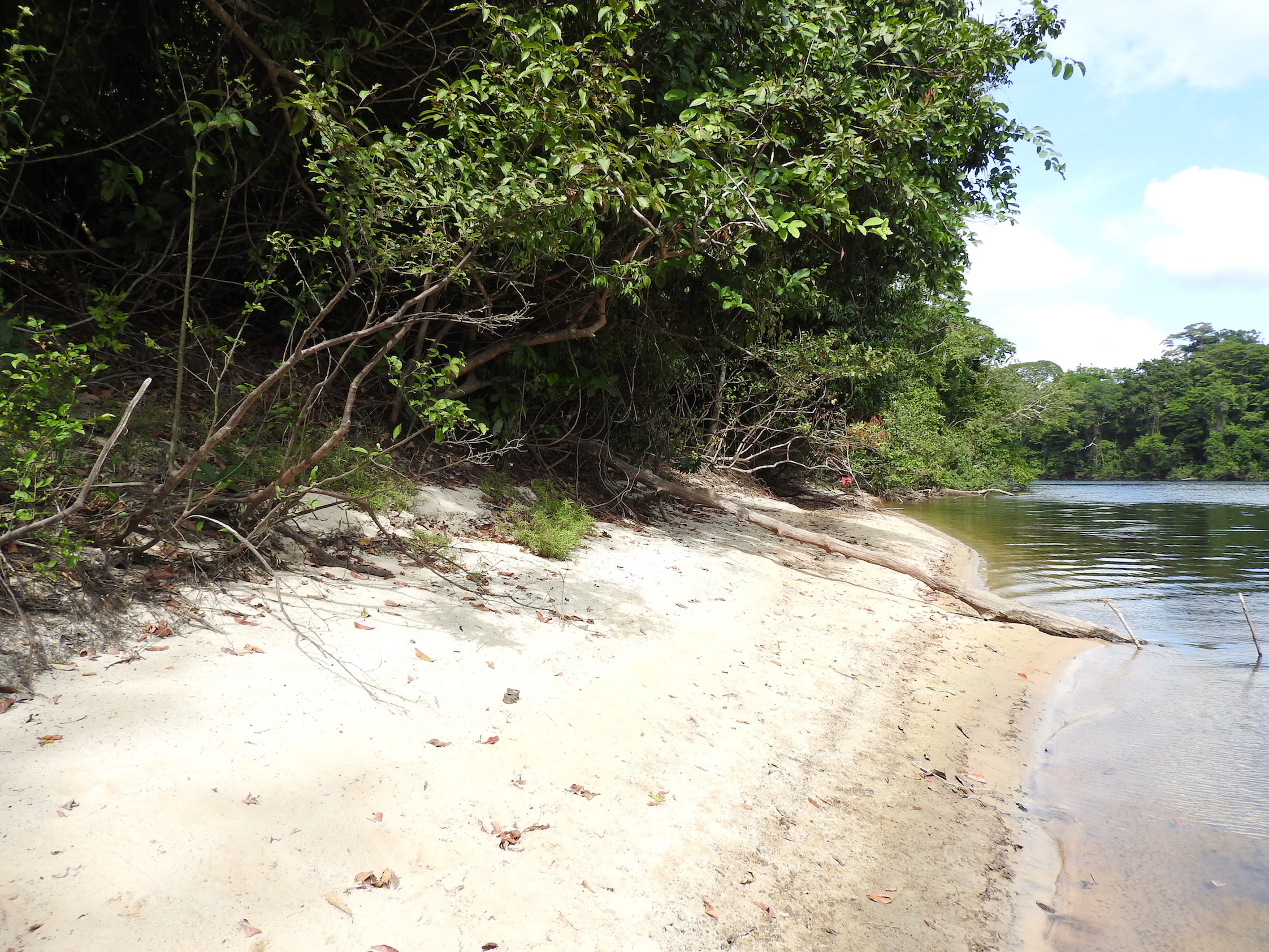 | B  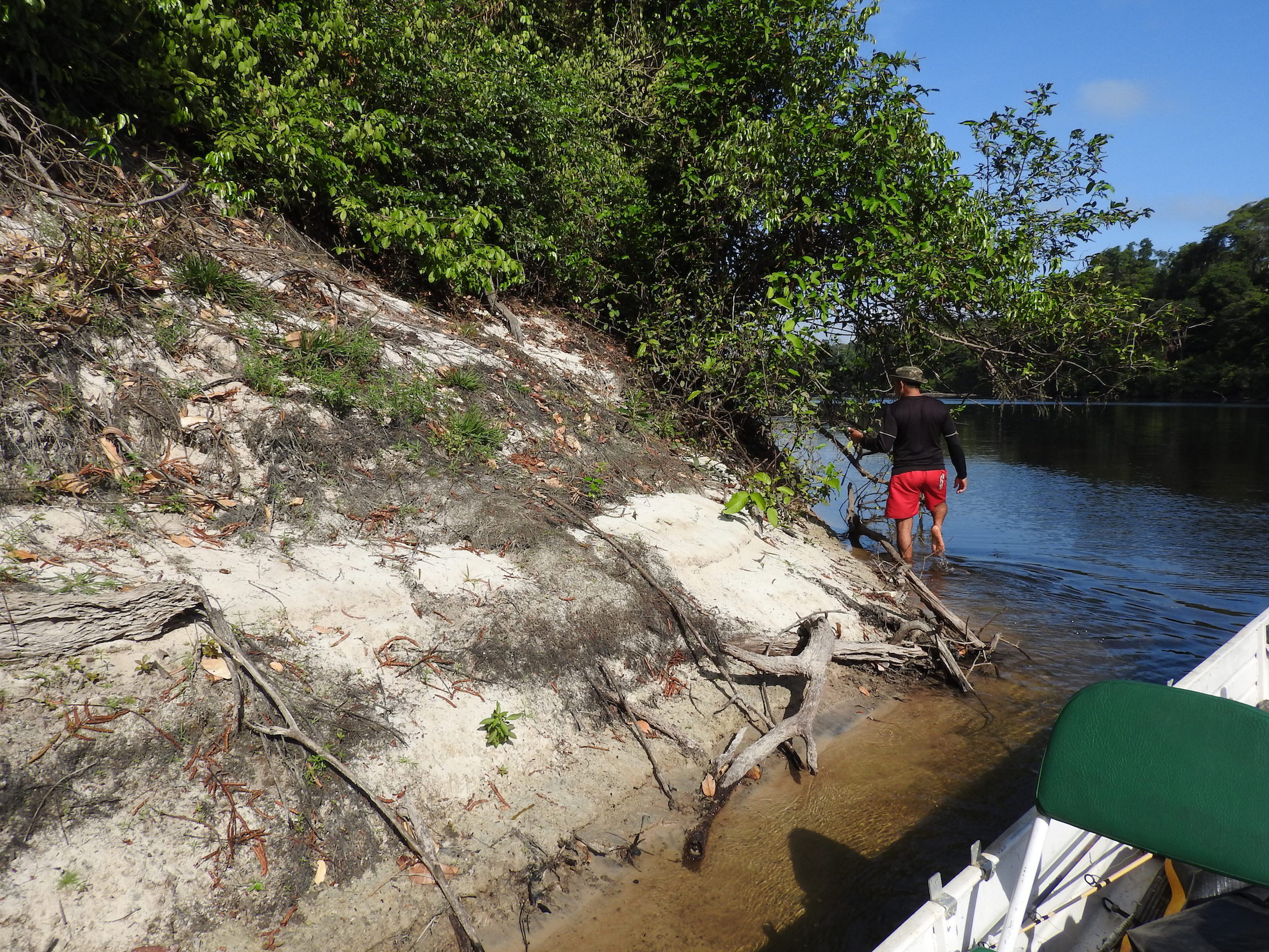 |
| --- | --- |
| C  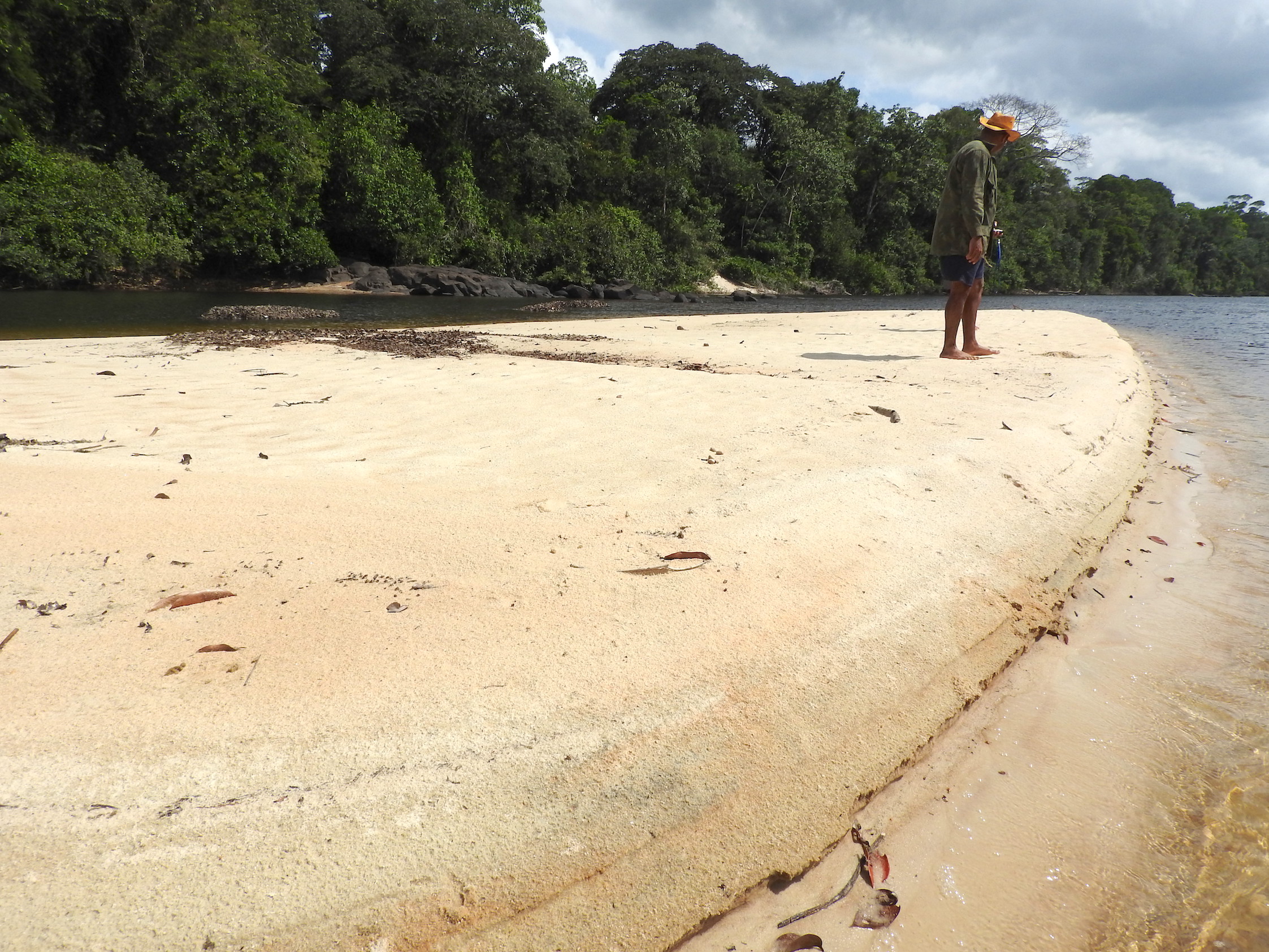 | D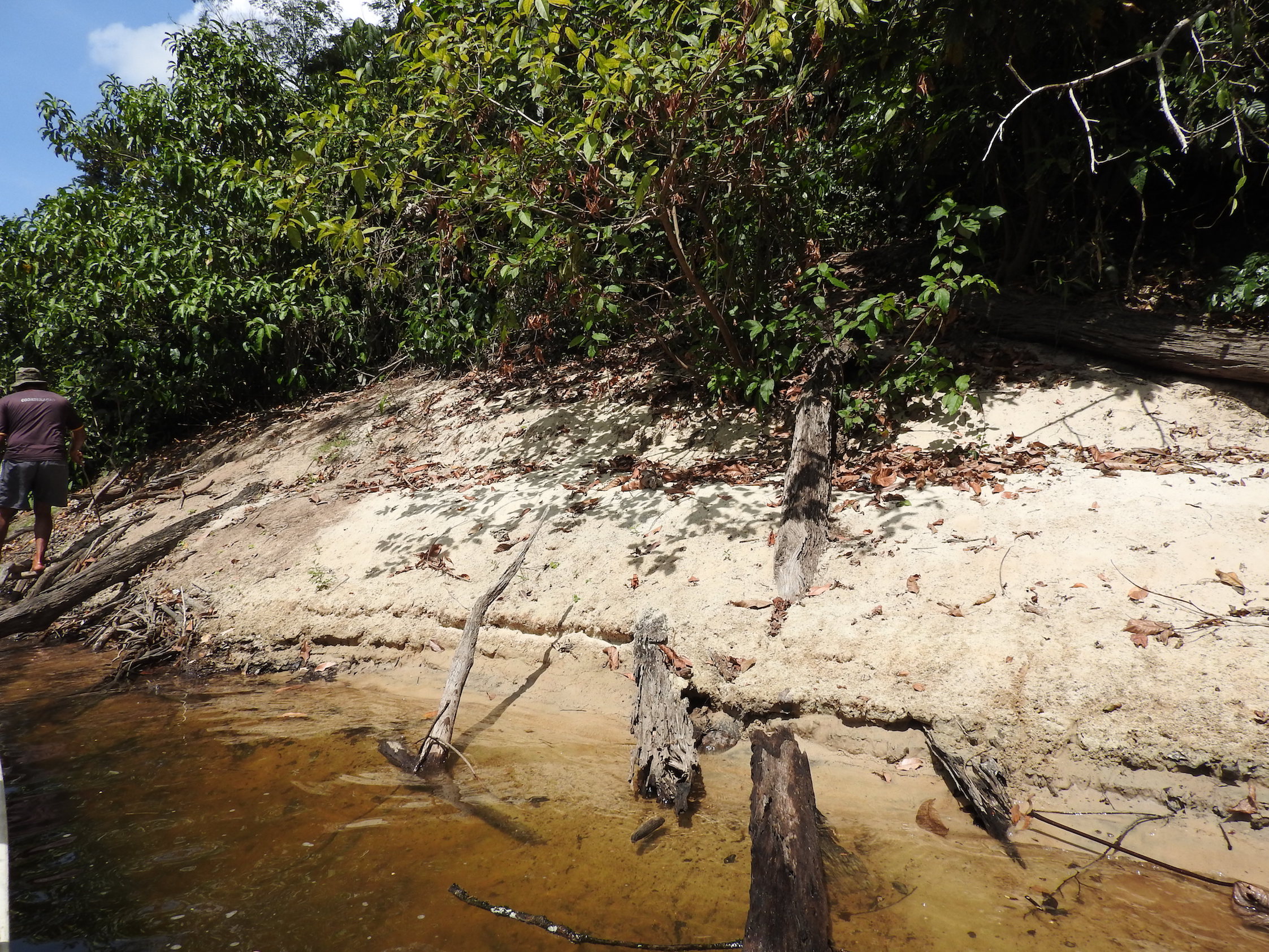 |
| E  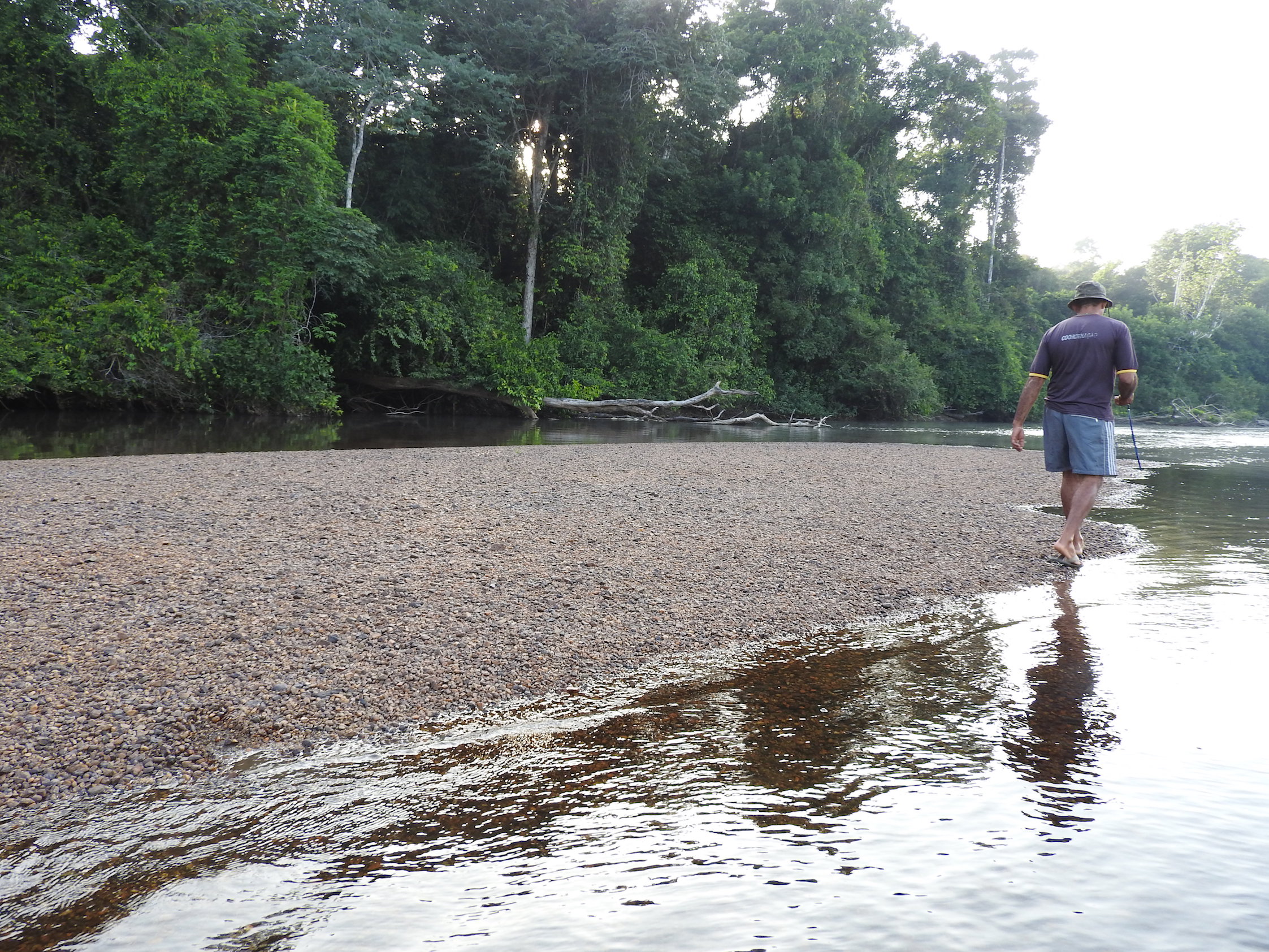 | F  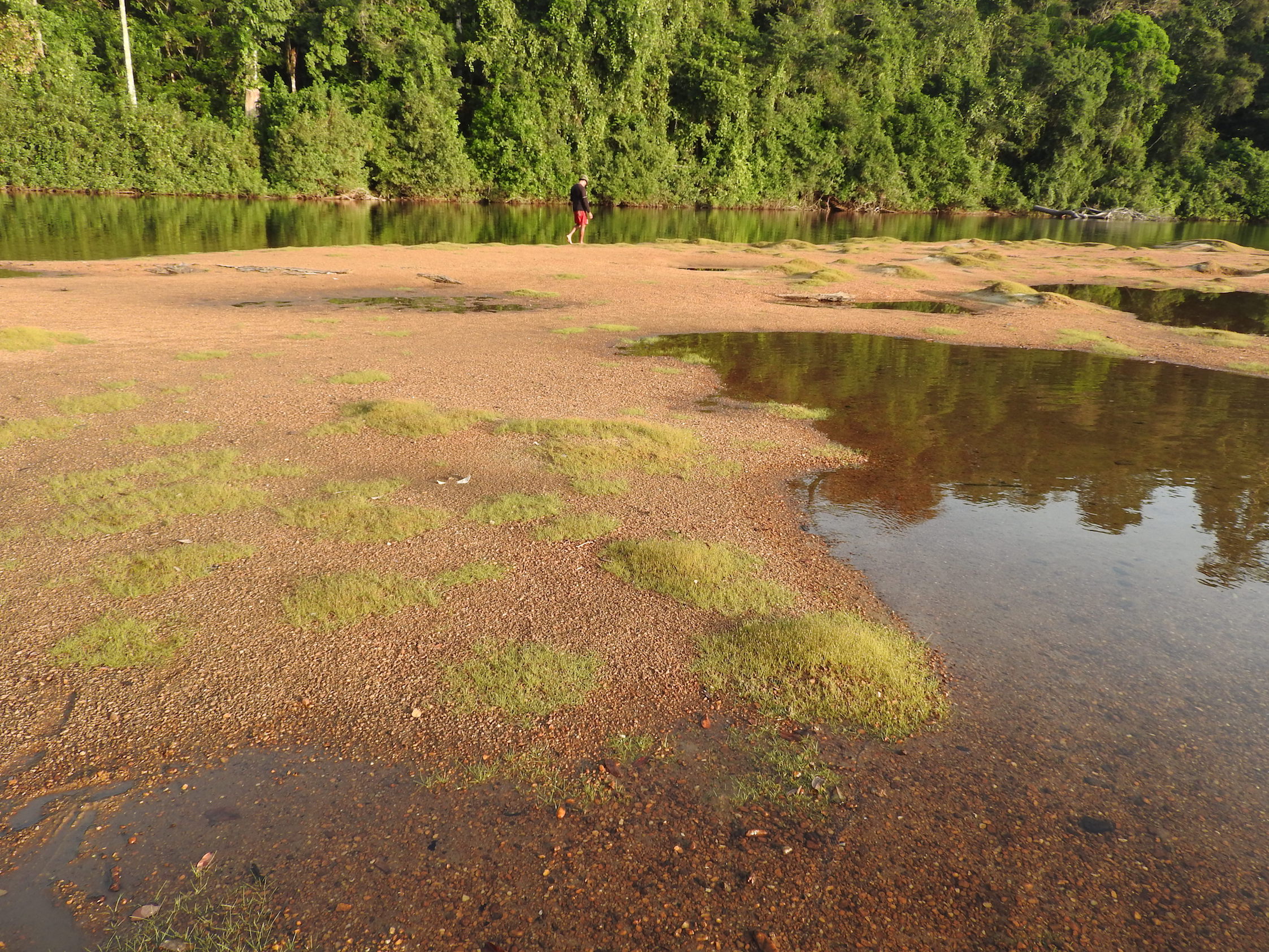 |
