# Supplementary material for: Beyond harm’s reach? Submersion of river turtle nesting areas and implications for restoration actions after Amazon hydropower development
Source: PeerJ. 2018 Jan 8;6:e4228. doi: 10.7717/peerj.4228 (PMC5764030; doi:10.7717/peerj.4228)
Supplement: Table S2 — Dataset used in Fig. 3. [file peerj-06-4228-s003.docx]

Table S2 Coordinates of submerged nest-area center points

| ID site | Area (m^2^) | Nesting | Distance to Dam (m) | longitude | latitude |
| --- | --- | --- | --- | --- | --- |
| 1 | 361 | Actual | 2088 | -51.36758 | 0.75439 |
| 2 | 48 | Potential | 2220 | -51.36800 | 0.75330 |
| 3 | 132 | Potential | 2188 | -51.36781 | 0.75329 |
| 4 | 11 | Potential | 2138 | -51.36682 | 0.75304 |
| 5 | 15 | Potential | 2138 | -51.36661 | 0.75301 |
| 6 | 66 | Potential | 2990 | -51.37080 | 0.74751 |
| 7 | 438 | Potential | 2990 | -51.36777 | 0.74369 |
| 8 | 55 | Potential | 3040 | -51.36854 | 0.74424 |
| 9 | 502 | Actual | 3140 | -51.37054 | 0.74520 |
| 10 | 41 | Potential | 3190 | -51.37087 | 0.74517 |
| 11 | 6 | Potential | 3190 | -51.37092 | 0.74512 |
| 12 | 16 | Potential | 3190 | -51.37080 | 0.74495 |
| 13 | 77 | Potential | 3240 | -51.37082 | 0.74413 |
| 14 | 640 | Potential | 3140 | -51.36905 | 0.74350 |
| 15 | 220 | Actual | 3040 | -51.36773 | 0.74259 |
| 16 | 374 | Potential | 3090 | -51.36821 | 0.74263 |
| 17 | 13 | Potential | 3190 | -51.36898 | 0.74228 |
| 18 | 438 | Potential | 3090 | -51.36710 | 0.74162 |
| 19 | 292 | Potential | 3140 | -51.36809 | 0.74159 |
| 20 | 730 | Actual | 3140 | -51.36825 | 0.74182 |
| 21 | 67 | Actual | 3440 | -51.37253 | 0.74355 |
| 22 | 101 | Potential | 3540 | -51.37331 | 0.74263 |
| 23 | 175 | Potential | 3640 | -51.37363 | 0.74187 |
| 24 | 1581 | Potential | 3711 | -51.37331 | 0.74065 |
| 25 | 85 | Actual | 3761 | -51.37363 | 0.74033 |
| 26 | 208 | Potential | 3761 | -51.37364 | 0.74009 |
| 27 | 31 | Potential | 3761 | -51.37438 | 0.74077 |
| 28 | 297 | Potential | 3861 | -51.37510 | 0.74034 |
| 29 | 103 | Potential | 3961 | -51.37594 | 0.74007 |
| 30 | 304 | Actual | 4061 | -51.37649 | 0.73970 |
| 31 | 1089 | Actual | 4111 | -51.37739 | 0.73948 |
| 32 | 95 | Actual | 4211 | -51.37816 | 0.73941 |
| 33 | 592 | Potential | 4261 | -51.37822 | 0.73866 |
| 34 | 371 | Actual | 4061 | -51.37505 | 0.73726 |
| 35 | 89 | Potential | 4161 | -51.37560 | 0.73692 |
| 36 | 544 | Potential | 4211 | -51.37626 | 0.73692 |
| 37 | 119 | Potential | 4261 | -51.37714 | 0.73725 |
| 38 | 247 | Potential | 4261 | -51.37726 | 0.73709 |
| 39 | 78 | Potential | 4311 | -51.38055 | 0.74093 |
| 40 | 293 | Potential | 4361 | -51.37861 | 0.73750 |
| 41 | 97 | Actual | 4461 | -51.37975 | 0.73737 |
| 42 | 744 | Potential | 5006 | -51.38492 | 0.73667 |
| 43 | 445 | Potential | 5756 | -51.39053 | 0.73236 |
| 44 | 214 | Potential | 5919 | -51.39147 | 0.73174 |
| 45 | 229 | Potential | 6019 | -51.39158 | 0.73017 |
| 46 | 574 | Potential | 6219 | -51.39435 | 0.73160 |
| 47 | 135 | Potential | 6219 | -51.39410 | 0.73104 |
| 48 | 47 | Potential | 6219 | -51.39497 | 0.73205 |
| 49 | 95 | Potential | 6319 | -51.39552 | 0.73158 |
| 50 | 15 | Potential | 6319 | -51.39429 | 0.72927 |
| 51 | 63 | Potential | 6319 | -51.39403 | 0.72893 |
| 52 | 108 | Potential | 7069 | -51.40048 | 0.72612 |
| 53 | 110 | Potential | 7069 | -51.40007 | 0.72603 |
| 54 | 53 | Potential | 7119 | -51.40183 | 0.72797 |
| 55 | 66 | Potential | 7802 | -51.40590 | 0.72268 |
| 56 | 68 | Potential | 7952 | -51.40621 | 0.72077 |
| 57 | 2 | Potential | 8002 | -51.40637 | 0.72070 |
| 58 | 9 | Potential | 8002 | -51.40639 | 0.72063 |
| 59 | 347 | Potential | 8002 | -51.40685 | 0.72084 |
| 60 | 103 | Potential | 8052 | -51.40723 | 0.72048 |
| 61 | 1973 | Potential | 8052 | -51.40752 | 0.72086 |
| 62 | 1873 | Actual | 9260 | -51.41628 | 0.71801 |
| 63 | 27 | Potential | 9160 | -51.41554 | 0.71743 |
| 64 | 1188 | Potential | 10448 | -51.42646 | 0.71701 |
| 65 | 324 | Potential | 10569 | -51.42720 | 0.71720 |
| 66 | 390 | Potential | 11230 | -51.43306 | 0.71587 |
| 67 | 382 | Potential | 10959 | -51.43016 | 0.71486 |
| 68 | 3 | Potential | 14209 | -51.45513 | 0.71959 |
| 69 | 7 | Potential | 14209 | -51.45521 | 0.71976 |
| 70 | 2 | Potential | 14259 | -51.45527 | 0.71989 |
| 71 | 57 | Actual | 14259 | -51.45537 | 0.72015 |
| 72 | 82 | Potential | 15120 | -51.45752 | 0.72769 |
| 73 | 198 | Potential | 15220 | -51.45783 | 0.72846 |
| 74 | 18 | Potential | 15253 | -51.45805 | 0.72894 |
| 75 | 43 | Potential | 15646 | -51.45578 | 0.73239 |
| 76 | 78 | Potential | 15810 | -51.45615 | 0.73328 |
| 77 | 116 | Potential | 15860 | -51.45642 | 0.73380 |
| 78 | 892 | Actual | 15942 | -51.45675 | 0.73453 |
| 79 | 56 | Potential | 15992 | -51.45680 | 0.73526 |
| 80 | 1512 | Actual | 16449 | -51.45728 | 0.73899 |
| 81 | 22 | Potential | 17841 | -51.46714 | 0.74061 |
| 82 | 137 | Potential | 17701 | -51.46650 | 0.74359 |
| 83 | 219 | Potential | 17741 | -51.46691 | 0.74345 |
| 84 | 134 | Potential | 17741 | -51.46721 | 0.74332 |
| 85 | 18 | Potential | 17912 | -51.46858 | 0.74246 |
| 86 | 40 | Potential | 17962 | -51.46884 | 0.74227 |
| 87 | 403 | Potential | 18012 | -51.46917 | 0.74205 |
| 88 | 212 | Potential | 19943 | -51.48633 | 0.74448 |
| 89 | 40 | Potential | 25369 | -51.53225 | 0.73666 |
| 90 | 2515 | Potential | 27015 | -51.53932 | 0.72304 |
| 91 | 98 | Potential | 29211 | -51.55775 | 0.73393 |
| 92 | 483 | Actual | 31160 | -51.57144 | 0.73361 |
| 93 | 916 | Potential | 31469 | -51.57388 | 0.73224 |
| 94 | 699 | Potential | 31849 | -51.57634 | 0.73079 |
| 95 | 5459 | Actual | 34487 | -51.58403 | 0.75102 |
| 96 | 66 | Potential | 34896 | -51.58412 | 0.75484 |
| 97 | 742 | Actual | 35722 | -51.58161 | 0.76184 |
| 98 | 56 | Potential | 45573 | -51.60703 | 0.83005 |
| 99 | 121 | Potential | 45943 | -51.60684 | 0.83317 |
| 100 | 39 | Potential | 45943 | -51.60703 | 0.83328 |
| 101 | 11 | Potential | 52898 | -51.61798 | 0.87980 |
| 102 | 795 | Potential | 52931 | -51.61762 | 0.88005 |
| 103 | 10 | Potential | 53516 | -51.61423 | 0.88278 |
| 104 | 213 | Actual | 53516 | -51.61451 | 0.88288 |
| 105 | 520 | Actual | 57351 | -51.60276 | 0.90684 |
| 106 | 301 | Potential | 44098 | -51.61045 | 0.81737 |
